# The Efficacy of Transverse Abdominis Plane Block for Postoperative Analgesia in Laparoscopic Hysterectomy: A Randomized Prospective Study

**DOI:** 10.7759/cureus.34666

**Published:** 2023-02-06

**Authors:** Navya Mishra, Manisha Bhagat, Ekramul Haque

**Affiliations:** 1 Anaesthesiology, Rajendra Institute of Medical Sciences, Ranchi, IND

**Keywords:** tramadol, analgesia, hysterectomy, laparoscopic, ultrasound, tap block

## Abstract

Background and aim: Postoperative pain is an inevitable acute pain for which a multimodal analgesic approach is required. The aim of this study was to quantify and compare the efficacy of transverse abdominis plane (TAP) block as a postoperative analgesic for patients undergoing laparoscopic hysterectomies versus intravenous opioid analgesics.

Method: Sixty female patients were enrolled and randomized into two groups following a computer-generated sequence of numbers. In group T (n=30) patients received an ultrasound-guided bilateral TAP block with 20 of 0.375% levobupivacaine on each side immediately after surgery. Patients in group O (opioid group, n = 30) received intravenous (i.v.) tramadol (100mg) immediately after surgery. Rescue analgesic (inj. tramadol 50mg) i.v. bolus given in both groups if visual analog scale (VAS) >4. In the postanesthesia care unit (PACU), the vital signs, episodes of nausea, vomiting, and VAS score of each patient were recorded every two hours for the first 24 hours.

Result: Total consumption of rescue analgesic (inj. tramadol 50 mg i.v.) during the first 24 hours was significantly higher in group O (186.47+37.48mg) than in group T (107.28+26.34mg). No significant difference was observed in intraoperative vital parameters (HR, NIBP, SPO_2_). The VAS scores were significantly low in group T. Incidence of postoperative nausea and vomiting (PONV) was significantly higher in group O (13 out of 28 patients) than in group T (five out of 28 patients) with P value =0.043.

Conclusion: Our study indicated bilateral ultrasound-guided TAP block is a good alternative to opioids for postoperative analgesia.

## Introduction

Postoperative pain is an inevitable pain of acute nature. Therefore, pre-emptive approaches should be incorporated into the treatment plan. Although it occurs commonly and can be predicted easily, treatment of postoperative pain is typically inadequate [1]. Post-laparoscopic surgery pain is multifactorial and includes somatic, visceral, and referred etiologies [1,2]; therefore a multimodal therapeutic approach is desirable.

The transverse abdominis plane (TAP) block is a regional block anesthetizing the nerves (T10 to L1) hence covering the lower abdominal wall [3]. Post-laparoscopic hysterectomy pain has two components: somatic and visceral. Somatic pain has two subdivisions, cutaneous and deep, Cutaneous pain originates from nociceptors within the abdominal wall and is transmitted via T10-L1 spinal nerves running laterally in the abdominal wall in between transverse abdominis and internal oblique muscle layers [1,4]. Visceral uterine nociceptive pain is transmitted via the afferent nerve stimuli ascending through the inferior hypogastric plexus which enters the spinal cord (T10-L1 nerves) [1,4].

The aim of this study was comparison and quantification of efficacy of TAP block as a postoperative analgesia technique for patients undergoing laparoscopic hysterectomies versus intravenous opioid analgesics with the primary objective being a multimodal approach for postoperative analgesia and secondarily to reduce the use of narcotics for postoperative analgesia.

## Materials and methods

This prospective randomized study was conducted in the department of Anaesthesia, Rajendra Institute of Medical Sciences (RIMS), Ranchi, after obtaining approval from Institutional Ethical Committee RIMS memo number 26, dated 10.02.2022 (IRB). Written informed consent was obtained from each patient. Considering a 95% confidence interval and 80% power in previous studies [3,4], the sample size was determined as 30 in each of the two groups. A total of 60 female patients 30 to 64 years of age undergoing laparoscopic hysterectomy, of American Society of Anesthesiologists (ASA) grade I, II, and body mass index (BMI) <30kg/m^2^ were enrolled in this randomized prospective study. Patients who refused to give consent or were not belonging to age (30-64 years) or ASA grade I, II, or BMI >30kg/m^2^, or any local skin infection in the lower abdominal region were excluded from our study.

**Figure 1:**
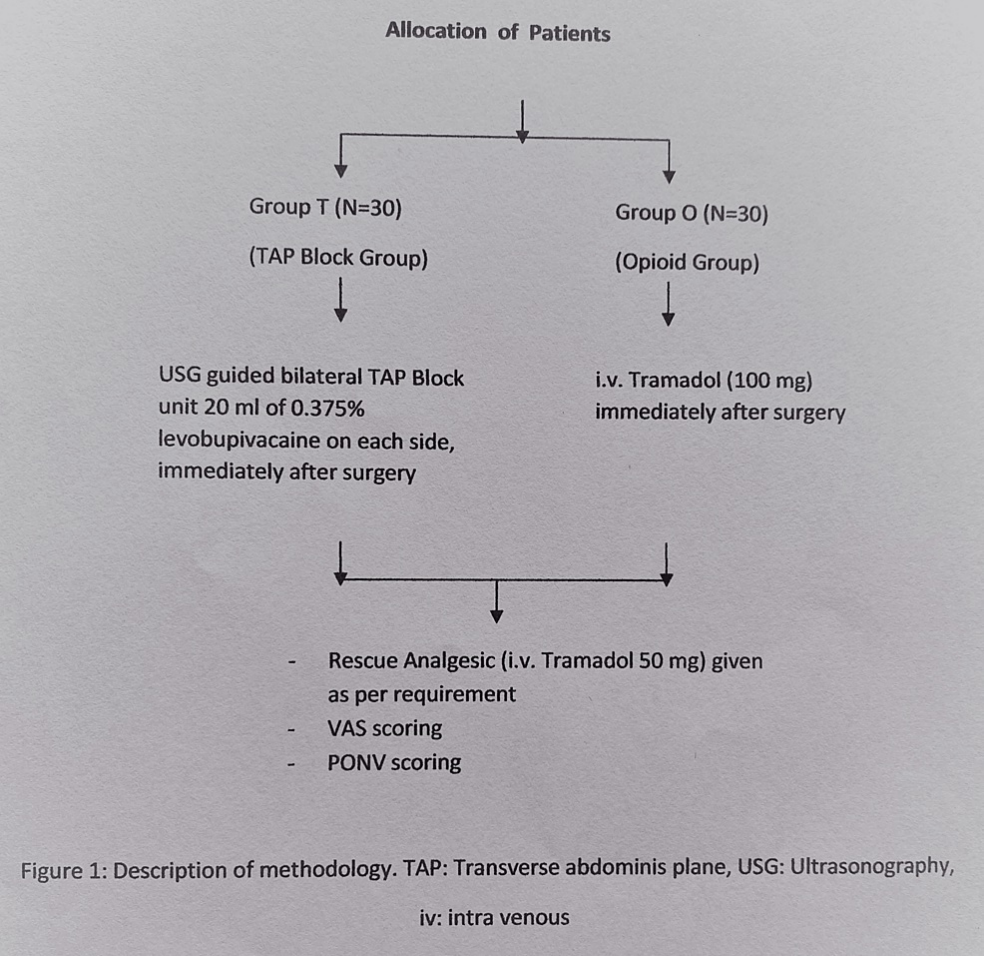
Flow chart describing the methodology VAS: visual analog scale; PONV: postoperative nausea and vomiting

After the arrival of the patient in the surgical ward, written informed consent was taken, and all patients were randomized into two groups using numbers generated using computer software. For each randomized patient in the operating room (OR), the anaesthesiologist took the corresponding sealed envelope from a folder, indicating the treatment assigned to the patient. Group T (n=30) patients received ultrasound-guided bilateral TAP block and after negative aspiration 40 mL of 0.375% levobupivacaine (20 mL for each side) was administered immediately after completion of surgery followed by rescue analgesic (injection (inj.) tramadol 50mg intravenous (i.v.) bolus) if visual analog scale (VAS) score >4 for the first postoperative 24 hours. Patients in group O (n = 30) were administered i.v. tramadol (100mg) immediately after surgery for postoperative analgesia followed by rescue analgesic (inj. tramadol 50mg) i.v. bolus (Figure 1).

Before induction, all vital signs (NIBP, heart rate, SPO_2_) were recorded. An infusion of crystalloids (ringer lactate) was administered to every patient throughout the surgery. Premedication was done by injection of Glycopyrrolate (0.02mg/kg), and inj. Midazolam (1mg). In both the groups, for analgesia inj. Fentanyl (2mcg/kg) was administered to all patients. General anesthesia was induced using inj. propofol (2mg/kg) and endotracheal intubation was done following muscle relaxation obtained by administrating inj. vecuronium (0.08mg/kg) to all the patients. General anesthesia was maintained by Air: O_2_ 50:50, isoflurane (0.8%), and inj. vecuronium in maintenance dose (0.05mg/kg). Once the uterus was removed and closure started bilateral TAP block was given in patients belonging to group T. Anatomical marking was done (patient in supine position) between the costal margin and the iliac crest at the midaxillary line for the placement of linear transducer (8-12hz), and the three layers of anterolateral abdominal wall were observed. A 25-gauge spinal needle was then inserted in the plane between the internal oblique and transverse abdominis muscle; after confirming the location with ultrasound and negative aspiration (to rule out any injury to blood vessels) 20ml of 0.375% levobupivacaine was injected into each side, whereas patients in group O (n = 30) received i.v. tramadol (100mg) immediately after removal of uterus for postoperative analgesia followed by rescue analgesic (inj. tramadol 50mg i.v. bolus). The anesthesiologist performing the block was unblinded to randomization. An observer blinded to group allocation was present outside the OT during the induction of anesthesia and block application and called back to the OT at the beginning of surgery for data collection.

All vitals (BP, HR, RR, and SPO_2_) were recorded at regular intervals of 10 minutes during surgery and after extubation. After extubation, patients of both groups were shifted to the postanesthesia care unit (PACU) once they started responding to verbal orders and performing a five-second head lift. All vital signs, episodes of nausea/vomiting, and VAS scores of each patient were recorded every two hours for the first 24 hours post-surgery. Pain severity assessment was done using a Numeric Rating Scale VAS score of 0 to 10.

Statistical analysis

The difference in tramadol consumption in the first postoperative 24 hours between group T and group O was the primary endpoint of our study. Secondary outcomes were pain measurements (VAS score from 0 to 10) during the first 24 hours postoperatively, times to surgical ward discharge, and postoperative nausea and vomiting (PONV) incidence.

Different statistical aggregates like continuous variables were expressed as mean ± standard deviation to analyze numerical parameters. An appropriate statistical method was used to determine the significance of the difference between comparisons. Student's t-test for difference between the mean of different data employed. For comparison of the incidence of side effects in the two groups, the chi-square test was applied. Differences between various parameters among different groups were considered significant if the p-value was < 0.05.

## Results

A total of 60 patients were enrolled in this study out of which patients 12, 19, and 32 received other analgesics which were not accepted in the protocol. In patients 7 and 48 intraoperative conversions to open surgery was done and were therefore not included in our study. Therefore, the total number of patients included in the study was 28 in group T and 28 in group O. The demographic characteristics such as patient’s age, BMI, or ASA score of both the groups when compared showed no significant difference (Table 1). Total consumption of rescue analgesic (inj. tramadol 50 mg i.v.) during the first 24 hours was significantly higher in group O (186.47+37.48mg) than in group T (107.28+26.34mg) (Table 2). The duration of postoperative analgesia ranged from 120 to 418 minutes in group T and from 74 to 164 minutes in group O, therefore significant difference (P<0.010) was observed in the mean duration of postoperative analgesia of group T (298.34+67.02 minutes) and group O (107.68+27.28 minutes). Furthermore, the incidence of PONV was significantly higher in group O (13 out of 28 patients) than in group T (five out of 28 patients) with a P value <0.05 (Table 2). The mean duration of anesthesia and surgery in the TAP block group was (124+9 minutes) whereas in group O it was (118+7 minutes) showing no significant difference (Table 3). The difference in intraoperative vital parameters (HR, NIBP, SPO_2_) in both groups was insignificant (Table 3). On comparison, the VAS scores showed significant statistical differences showing much lower values in group T than in group O at 2, 4, 6, 12, 18, and 24h respectively although the difference was insignificant at the one-hour postoperative period (Table 4).

**Table 1:** Demographic and pre-operative parameters Values are presented as numbers or mean + SD. ASA: American Society of Anaesthesiologists, bpm: beat per minute, MAP: mean arterial pressure

Parameters	Group T ( n=28)	Group O (n=28)	P value
Age (years)	37.28 ± 14.63	35.32 ± 15.09	0.920
Weight (Kg)	50.68 ± 7.62	49.82 ± 4.68	0.600
Height (cm)	152.88 ± 5.90	153.46 ± 5.60	0.380
ASA status (I/II)	18/10	20/10	0.760
Preoperative heart rate (bpm)	82.34 ± 6.88	79.32 ± 6.35	0.340
Preoperative MAP (mmHg)	90.14 ± 5.78	92.1 ± 8.32	0.140

**Table 2:** Total consumption of rescue analgesic Values are presented as numbers or mean + SD. VAS: visual analog scale

Parameters	Group T (n=28)	Group O (n=28)	P value
Time to first rescue analgesic at VAS >4 (mins)	298.34 ± 67.02 (min)	107.68 ± 27.28 (min)	P <0.010
Total consumption of rescue analgesic	107.28 ± 26.34 (mg)	186.47 ± 37.48 (mg)	P <0.05
Incidence of postoperative nausea and vomiting	5/28	13/28	P <0.05

**Table 3:** Mean duration of anesthesia and surgery Values are presented as numbers or mean + SD. NIBP: non-invasive blood pressure

	Group T (n=28)	Group O (n=28)	P value
Mean duration of anesthesia and surgery	124 ± 9 (minutes)	118 ± 7 (minutes)	0.364
Heart rate	78.39 ± 6.25	79.20 ± 6.35	0.860
NIBP	91.20 ± 6.88	90.34 ± 6.35	0.340
SPO_2_	99.20 ± 0.88	99.64 ± 0.35	0.360

**Table 4:** VAS score mean in the postoperative period Values are presented as numbers or mean + SD. VAS: visual analog scale

Time (h)	Group T (n=28)	Group O (n=28)	P value
1	0.12 ± 0.04	0.43 ± 0.76	0.054
2	0.14 ± 0.04	1.43 ± 0.76	< 0.01
4	0.38 ± 0.072	3.62 ± 1.09	< 0.01
6	1.68 ± 0.71	3.84 ± 0.76	<0.05
12	3.74 ± 0.78	2.06 ± 0.47	<0.05
18	3.47 ± 0.38	2.92 ± 0.56	0.048
24	3.04 ± 0.46	2.98 ± 0.72	0.049

## Discussion

In today’s era, a multimodal postoperative approach of good quality analgesia is required to overcome acute postoperative pain and also to minimalize postoperative adverse effects. Opioids such as tramadol and morphine generally used for postoperative analgesia cause significant adverse effects such as nausea, vomiting, and sedation. Therefore, well-planned analgesia needs to be introduced to reduce the incidence of side effects and opioid consumption and to ensure patient comfort and early mobilization and discharge in the postoperative period [5]. Peripheral nerve blocks play an essential role in the multimodal approach to postoperative analgesia. The TAP block was first described by Rafi in 2001 [6]. Ultrasound-guided TAP block was first described by Hebbard et al. in 2007 [7]. In our study the VAS score recorded was much less in the TAP block group than in the opioid group. Similar observations were made in previous studies [8,9]. Like previous studies in our study also the need for additional analgesics was less in the TAP block group than in the opioid group [8]. Like the previous study [8], in our study also the time to the first analgesic in the postoperative period was significantly greater in group T than in group O as TAP block provided a longer duration of postoperative analgesia. Most of the previous studies used morphine for postoperative analgesia for comparison [10,11], however, in our study, we have used tramadol due to its easy availability in our institute [8,12]. Tramadol has a higher incidence of nausea and vomiting than morphine [13]. Similarly, Toker et al. in their study also used tramadol and found that tramadol consumption was significantly less in the TAP block than in the opioid group. Likewise in our study also the tramadol consumption was much less in the TAP block group than in the opioid group [8]. Due to reduce consumption of tramadol in the TAP block group our study also reported significantly less incidence of nausea and vomiting in the TAP block group than in group O. Similar observation was made by Toker et al. in their study [8]. However previous studies in which morphine was used for postoperative analgesia, no significant difference in the incidence of PONV was reported between the two groups [10,11]. Similar to previous studies, in our study also, we have 20 ml of 0.375% bupivacaine on each side in the bilateral TAP block group [8]. Observations of our study clearly indicate that TAP block reduces postoperative pain and decreases consumption of opioids and associated side effects henceforth proving it to be a good choice for multimodal analgesia. No complications due to local anesthesia such as drug toxicity were reported in our study; likewise in previous studies also [8,9], no such complications were reported. The limitations of our study were the non-availability of PCA pumps in our institute and pain assessment done only for the first 24 hours postoperative period.

## Conclusions

Our study indicates that bilateral ultrasound-guided TAP block is a good alternative to opioids for postoperative analgesia and therefore can be considered as an important component of the multimodal analgesic approach. A multimodal approach of analgesia provides reduced postoperative pain, early mobilization of patients, and improves the scope of early hospital discharge for patients.
